# When is Deceptive Message Production More Effortful than Truth-Telling? A Baker’s Dozen of Moderators

**DOI:** 10.3389/fpsyg.2015.01965

**Published:** 2015-12-24

**Authors:** Judee K. Burgoon

**Affiliations:** Center for the Management of Information, Eller College of Management, University of ArizonaTucson, AZ, USA

**Keywords:** deception, cognitive effort, truth, deceptive message production, moderators of deception displays

## Abstract

Deception is thought to be more effortful than telling the truth. Empirical evidence from many quarters supports this general proposition. However, there are many factors that qualify and even reverse this pattern. Guided by a communication perspective, I present a baker’s dozen of moderators that may alter the degree of cognitive difficulty associated with producing deceptive messages. Among sender-related factors are memory processes, motivation, incentives, and consequences. Lying increases activation of a network of brain regions related to executive memory, suppression of unwanted behaviors, and task switching that is not observed with truth-telling. High motivation coupled with strong incentives or the risk of adverse consequences also prompts more cognitive exertion–for truth-tellers and deceivers alike–to appear credible, with associated effects on performance and message production effort, depending on the magnitude of effort, communicator skill, and experience. Factors related to message and communication context include discourse genre, type of prevarication, expected response length, communication medium, preparation, and recency of target event/issue. These factors can attenuate the degree of cognitive taxation on senders so that truth-telling and deceiving are similarly effortful. Factors related to the interpersonal relationship among interlocutors include whether sender and receiver are cooperative or adversarial and how well-acquainted they are with one another. A final consideration is whether the unit of analysis is the utterance, turn at talk, episode, entire interaction, or series of interactions. Taking these factors into account should produce a more nuanced answer to the question of when deception is more difficult than truth-telling.

Common sense tells us that lying should be more difficult than truth-telling. After all, the truth is ready-made; the lie must be invented. Ceteris paribus, more effort is involved in fabricating a falsehood than in accessing and producing a veridical account of something that is already stored in memory.

But common sense is not always the best teacher. There are many circumstances under which truth-telling imposes more challenges than deceiving. I therefore want to advance the hypothesis that the effort associated with deceiving vice truth-telling is a function of the characteristics of the communication event in force and that deeper analysis of critical elements of the communication process will bring more clarity to the issue of the cognitive effort associated with deceit. Although many such elements have been included as moderators in deception meta-analyses, their impact has not necessarily been attributed to cognitive (or emotional) exertion, and reliable empirical associations are few. A more coherent framework is therefore wanting.

## The Dominant Pattern

First let us consider the received wisdom that deception is more difficult than truth and some of the evidence that undergirds it. Numerous deception scholars have argued that deception is more effortful than truth-telling (e.g., Zuckerman et al., 1981; Miller and Stiff, 1993; Buller and Burgoon, 1996b; Vrij, 2000; Sporer and Schwandt, 2006). Empirical research has affirmed this view with evidence of measurable psycho-physiological indicators of arousal and stress (e.g., the wealth of research on the polygraph; see Gougler et al., 2011) as well as observable behavioral signs of performance decrements. Deceptive messages are often shorter, slower, and less fluent, with longer response latencies, averted gaze, temporary cessation of gestures and postural rigidity–all potential indicators of deceivers having to think hard (Goldman-Eisler, 1958; Vrij et al., 1996, 2006; Rockwell et al., 1997; Porter and ten Brinke, 2010; ten Brinke and Porter, 2012; Mullin et al., 2014).

That said, it is important to note that the mental machinations associated with deception need not be burdensome or uniformly so. As Buller and Burgoon (1996b) stated in a rejoinder to DePaulo et al. (1996):

…DePaulo et al. (1996) ascribe to us a highly cognitive view of deception, with deceptive episodes peopled by highly conscious, surveillant liars and equally vigilant, cunning receivers. This is an exaggerated characterization of our assumptions. We have taken some pains in IDT to argue that much sender and receiver activity during deceptive encounters, like other communicative encounters, can be goal driven and strategic yet largely automatic and “mindless” (see, e.g., Kellermann, 1992; Burgoon and Langer, 1995). We see deception running the gamut from the kinds of inconsequential white lies and evasions that populate daily discourse to the life-threatening kinds of fabrications and omissions that color international conflicts (Burgoon and Buller, 1996, pp. 320–321).

The activities involved in message production are familiar, routinized, overlearned. Mental processes can be activated without the sender necessarily having significant attentional resources diverted. This is especially likely in the dominant laboratory research paradigms, which entail telling harmless and inconsequential lies seldom lasting more than 1 min and addressing single incidents, factual matters, or likes-dislikes. In such cases, messages can be constructed on the fly and modified in response to emergent exigencies. Senders can tap into a host of memories and readily accessible schemas that enable rattling off a deceptive response. The division of labor between verbal and non-verbal components of messages further distributes the workload and reduces the call on cognitive resources. Moreover, if lies are about inconsequential matters, are at the behest of an investigator, and entail no adverse consequences, then any emotional overlay should also be attenuated.

That many forms of deception are “ready-made” does not invalidate that the other processes surrounding their use, form and potential consequences still impose more cognitive work on the sender than does a truthful message related to the same narrative. But the depiction of deceptive message production requires more sophisticated modeling. It is not a question of deception being *either* easier or more difficult than telling the truth. It can be both.

## A Baker’S Dozen Of Moderators

Here, then, toward a more nuanced, communication-oriented view, are a baker’s dozen of factors that should tip the scales in one direction or another. This non-exhaustive collection includes sender factors (i.e., ones that reside within the individual producing a message), message and communication context factors (i.e., ones related to the content and style of the message and to the communication context), relationship factors (i.e., ones inhering in the interpersonal relationship between sender and receiver) that should enable predictions of the circumstances under which deception will be more effortful, and scale of the measurement window under analysis. I illustrate many with evidence from our research program on interpersonal and mediated deception.

### Sender Memory Demands

Recent neuroscience research is corroborating what social scientists have suspected for a long time—that the more a lie activates different mental processes, the more mental taxation it imposes on a communicator. In their updated conceptualization of cognitive resource demands associated with (complex) lie production, Sporer and Schwandt (2007) incorporated newer models of working memory such that cognitive load extends beyond accessing details from memory and constructing non-contradictory messages to also activating autobiographical and executive memory functions.

Consider that compared to the truth-teller, who needs only to recall an actual state of affairs, the deceiver must not only access the true state of affairs but must engage executive memory to decide *if* to deceive, evaluate which forms of deception are more “acceptable” according to one’s moral code and choose among those options, conduct a cost-benefit calculus of the relative likelihood of success of alternative forms of deceit, fabricate the response itself, compare it to the truth for possible inconsistencies with known facts, check the deceit against a “plausibility” meter, gage the likelihood of suspicion or detection by the interlocutor, and then actually assemble the verbal and non-verbal components into a normal-appearing message that maximizes credibility, all the while suppressing inapt behaviors and cognitions.

Early explorations of brain functioning with fMRI confirmed that these activities have associated changes in brain activation such that different regions show increased activation during lies than truths (see, e.g., Spence et al., 2001; Ganis et al., 2003; Abe and Greene, 2014). In one such test, Spence et al. (2008) found that the ventrolateral prefrontal cortex (VLPFC) was preferentially activated to inhibit inappropriate and unwanted cognitions and responses when lying about embarrassing material. Using a different method, Mameli et al. (2010) found multiple networks in the prefrontal cortex involved in deceptive responding as well as longer reaction times when communicators responded deceptively relative to truthful responses at baseline. Ito et al. (2011, p. 126) similarly substantiated increased activity in a network of brain regions in the dorsolateral prefrontal cortex (plus longer response latencies) when remembering and reporting truthful and deceptive neutral and emotional events. The authors did not find a similar response during truth-telling, leading them to suggest that “there is an increase in the amount of conflict and higher cognitive control needed when falsifying the responses compared to responding truthfully.”

A recent meta-analysis (Christ et al., 2009) further established that lying is associated with multiple executive control processes, specifically working memory, inhibitory control, and task switching (i.e., interspersing truthful with deceptive details). Using their activation likelihood estimate method, the authors demonstrated quantitatively that eight of 13 regions and 173 deception-related foci are consistently more active for deceptive responses than for truthful ones.

These robust findings using varied approaches are strong evidence that deception summons memory processes that are more taxing than those associated with truth-telling. Thus, for the predominant research paradigms that have been used, and holding all other conditions constant, deception requires engagement of more cognitive (and/or emotional) resources than does truth-telling^1^.

### Sender Motivation, Incentives, and Consequences

This general pattern notwithstanding, three interrelated moderators that can alter this conclusion are motivation, incentives and consequences. Because motivation has often been manipulated through high monetary incentives or escaping adverse consequences, these three factors are operationally confounded. High motivation is thought to muster more effort, which can interfere with performance *or* improve it. The motivation impairment effect (MIE) asserts that motivation impairs non-verbal performance, thereby making lies more transparent, but also facilitates deceivers’ verbal performance (DePaulo and Kirkendol, 1989; Bond and DePaulo, 2006). Empirical findings have been fraught with inconsistencies. Burgoon and Floyd (2000), Burgoon et al. (2012), and Burgoon et al. (2015) have found both impairment and improvement of non-verbal and verbal performance among motivated deceivers engaged in consequential deception. Additionally, high-motivation *truth-tellers* (not deceivers) sometimes were most affected. Two meta-analyses (that omitted the aforementioned investigations) found high motivation affected liars and truth-tellers equally (Bond and DePaulo, 2006), and high-motivation lies were neither more nor less detectable than other lies (Hartwig and Bond, 2014).

If communicators have little to gain from deceiving or to lose from being caught, lying may pose little more challenge than truth-telling. Aside from the memory demands discussed above, small everyday lies such as fibs and white lies are easy to produce, can draw upon a cache of previously used utterances, and countenance no danger if detected. Lies that are likely to summon more cognitive resources are those that yield high pay-off if successful or that place the deceiver in serious jeopardy if uncovered (Porter and ten Brinke, 2010). In an analysis of real high-stakes deception, ten Brinke and Porter (2012) found that deceivers feigning distress over their missing children had difficulty faking sadness, leaked expressions of happiness, and were verbally more reticent and tentative. The authors ascribed these performance decrements partly to increased cognitive load. In high-consequence circumstances, however, truthful individuals may be equally distressed or motivated to succeed, so the difficulty of producing believable messages may be similar regardless of veracity.

The diverse results suggest that motivation is more complicated than presupposed and requires more “unpacking” of its relationship to cognitive effort. From a communication standpoint, motivation should follow social facilitation predictions, aiding overlearned behavior and interfering with less practiced behavior, up to a point beyond which emotional flooding should impair both verbal and non-verbal performance. Communicator skill and experience should dictate the threshold for performance deterioration.

### Discourse Genre

Language can be categorized according to genres, which are discourse forms that share similarities in their structure, style, content, intended audience, and context in which they occur. Different genres impose qualitatively different demands on deceivers and truth-tellers. A factual narrative or description, for example, comprises representational and verifiable features that need to be assembled into a cogent, plausible sequence, and supported by relevant details. Whereas truth-tellers are only limited by the acuity of their memory when relaying specifics of an event, deceivers not only must recall the true state of affairs, but must decide how much, if any, to tell. They must compare their alternative version to reality, edit the content and linguistic form, and assemble the elements into a believable chronology.

Comparatively, an opinion lacks verifiability and need not be accompanied by any supportive documentation. Deceivers can easily proffer indisputable conjectures and opinions when asked questions such as, “Who do you think may have stolen the money from the cash draw?” or “What should happen to the thief?”, whereas the thoughtful reflections of a truth-teller may require more effort.

Within interactive discourse genres are also variations in form. A face-to-face dialog carries different demands than a monolog or one-to-many speech. When engaged in conversation with another, interlocutors must fulfill multiple communication functions beyond message production itself. First, they must “read” the definition of the situation from contextual cues so as to know what kind of discourse and associated expectations are in force. Because ascertaining identities is usually a high priority, communicators must signal their self-identity (e.g., gender, ethnicity, race, personality), put forth a desired self-presentation, and size up others’ identities. As interactions unfold, they must formulate their own messages and decipher the messages and feedback from their interlocutor. They must also regulate their emotional expressions, exchange relational messages that define the relationship between sender and receiver (e.g., trusting, intimate, equal), perform turn-taking responsibilities, and monitor their own communication. Although human communicators perform these functions in a seemingly effortless fashion, the discourse form can magnify or alleviate some of the effort associated with them. For example, Burgoon et al. (2001) demonstrated that engaging in dialog compared to face-to-face monolog was more difficult initially, but over time, dialog eased the demands on deceivers who were able to share the turn-taking burden with their interlocutor, create a smooth interaction pattern by developing interactional synchrony, adapt to interlocutor feedback, and approximate normal communication patterns^2^.

Another genre, the interview, can also influence the cognitive burden on respondents. The question-answer structure adds predictability to who is supposed to talk when and what the content should be. Language can be borrowed from the interviewer’s questions, and questions can be repeated as a stalling technique. Even within interviews are notable differences: Relative to an open-ended, free-wheeling interview, a structured one that requires short-answer replies reduces the degrees of freedom of what can be said and allows deceivers to forecast what is coming next. Many deception experiments are of this latter brief-answer variety, which our research has shown produces substantially different behavioral and psycho-physiological responses than open-ended interview protocols (Burgoon et al., 2010).

The illustrative genres mentioned here point to the need to formulate deception-relevant taxonomies of genres so that predictions can be made as to which will intensify or diminish the cognitive effort required of sender and receiver.

### Form of Prevarication

Contrary to the claims of McCornack et al. (2014) that virtually all extant deception research bifurcates deception into bald-faced lies or bald-faced truths, and regards only those discourse options as worthy of scholarly investigation, most deception scholars recognize that deception includes a variety of forms. A sampling of research across the last five decades and across multiple disciplines has identified such forms of prevarication as white lies, altruistic lies, omissions, concealment, equivocation, evasions, exaggerations, strategic ambiguity, and impostership (see, e.g., Turner et al., 1975; Hopper and Bell, 1984; Miller and Stiff, 1993; Buller et al., 1994; Searcy and Nowicki, 2005; Ennis et al., 2008; Knapp, 2008). The type of prevarication being told will affect the cognitive resources required in its telling.

In his original formulation of information manipulation theory (IMT), McCornack (1997) proposed that deceptive discourse violates conversational implicatures along one or more of Grice’s (1989) four dimensions of cooperative discourse: quantity, quality, manner, and relation. Burgoon et al. (1996) proposed a similar set of five dimensions of information management: completeness (comparable to quantity), veridicality (comparable to quality), clarity (comparable to manner), relevance (comparable to relation), and personalism (see also Buller and Burgoon, 1996a). Under both conceptualizations, some forms of deceit such as omissions are more easily produced than others^3^.

Other times, truth-telling can be more difficult than deceit. Having to convey a “hard” truth to a patient dying of a terminal disease can levy more cognitive taxation than manufacturing a comparable falsehood that there is hope for recovery from the disease. A provocative line of research on whether people lie automatically or must decide to lie has also shown that when cheating offers a high probability of personal gain, people may be quicker to produce self-serving lies than truthful responses. In tempting situations, if a self-benefiting lie is easy to craft and little time is allowed for reflection, lying may be the more automatic response, whereas honesty may necessitate more hesitation, deliberation, and executive control (Shalvi et al., 2012; Tabatabaeian et al., 2015; see also Bereby-Meyer and Shalvi, 2015, for a review of supporting literature). When social bonds are made salient, people also produce lies more quickly that benefit their social group than lies that benefit only self (Shalvi and De Dreu, 2014).

In short, the type of prevarication (or truth) can be located on a continuum from easy to difficult, with cognitive effort for easy lies making them no more challenging than telling the truth.

### Expected Response Length

Different kinds of interactions have associated expectations about utterance length. Day-to-day conversations are typified by reciprocation of short turns at talk. Conversing deceivers may project that they can get away with very brief responses while still satisfying conversational expectations. A spouse’s query, “How was your day?” is not expected to produce a dissertation on all one’s trials and tribulations at work or home. A husband who skipped work to go gambling or a wife on an illicit tryst can safely reply with a breezy “fine.” Such brief lies and truths—the bread and butter of much deception research–may differ little in their demands on resources. More penetrating questions like, “Why couldn’t I reach you today when I called your cell four times?” require lengthier–and more demanding–accounts.

Standard interview protocols also have associated expectations about what response lengths suffice. Introspective questions require conjectural rather than factual responses, and their non-verifiability may attenuate the memory burden on deceivers. The behavioral analysis interview operates on the premise that innocent people will exhibit the Sherlock Holmes effect: In attempting to aid an investigation, innocent respondents may speculate more than deceivers and widen the pool of suspects. Comparatively, deceivers should minimize conjecture and avoid proposing other suspects for fear of narrowing the pool to themselves (Horvath et al., 2008). A cognitive interview, in which respondents are asked to retell an account from multiple vantage points (Fisher and Geiselman, 1992), requests increasing elaboration and details, something that is expected to be easier for truth-tellers than deceivers to accomplish over repeated retellings (see also Vrij and Granhag, 2012).

Generally, conversations have associated norms and expectations for what kinds of utterances will satisfy the Gricean maxims, and communicators are fairly adept at predicting and fulfilling those expectations. The degree of cognitive difficulty should correlate positively with response length and how much the deceptive response deviates from expected form (with exceptions that can be anticipated in advance).

### Sanctioning of Deceit

Most laboratory research involves deceit that is sanctioned by the experimenter rather than being chosen voluntarily by the perpetrator (Frank and Feeley, 2003). The alternative of allowing research participants to choose whether to lie or not creates a confound in that only skillful liars and those with an honest-appearing demeanor may choose to lie (Levine et al., 2010). Apart from experimenter-instigated deceit differing behaviorally from that chosen of a deceiver’s own volition (Sporer and Schwandt, 2007; Dunbar et al., 2013), the implication outside the laboratory is that deception will vary substantially in form and difficulty as a function of sanctioning and communicator skill (see also IDT regarding communicator skill).

That said, choice and skill may not completely alleviate the added cognitive work associated with deceit. Spence et al. (2008) designed an fMRI experiment in which deceivers could choose to comply or defy an experimenter’s request to divulge embarrassing secrets. Results revealed lying activated the VLPFC even under free choice. At the most fundamental level of brain functioning, then, lying still exercises a main effect on cognitive processing.

### Communication Medium

The medium of communication itself also influences the degree of cognitive difficulty associated with lying. IDT’s first proposition states, “*Context features of deceptive interchanges systematically affect sender and receiver cognitions and behaviors; two of special importance are the interactivity of the communication medium and the demands of the conversational task”* (Burgoon and Buller, 2015). To the extent that deceivers are interacting synchronously and with all audiovisual modalities available to receivers (e.g., face-to-face, computer-mediated communication, teleconferencing), there are more communication functions to which cognitive resources must be devoted. When modalities are more limited–such as voice or chat–and asynchronous—more resources can be distributed among fewer aspects of message production and with less time press.^4^ Consistent with this reasoning, participants in a mock theft experienced the least anxiety and cognitive load when interacting via text, were the most aroused and exercised the most behavioral control when interacting face-to-face, and reported the most cognitive effort when interacting via an unfamiliar audio format (Burgoon et al., 2004; Burgoon, 2015). Thus, leaner and non-interactive media should attenuate cognitive effort.

### Preparation

This construct subsumes many related variables—advance thought, planning, rehearsal, or editing. Extemporaneous or unscripted discourse is produced in real time; planned, rehearsed, or edited discourse entails some intervening time interval between the deliberation and construction of a message and its ultimate delivery. Such *ex ante* preparation may be experimentally manipulated, as in a classic interviewing investigation by O’Hair et al. (1981), or it may be prompted by high-stakes circumstances such as queries about fraudulent financial reporting: “…individuals may, for example, prepare extensively before speaking to lower the cognitive burden that can accompany deception, or may undergo voice training in an attempt to sound vocally like the antithesis of someone engaging in deception” (Burgoon et al., 2015, p. 2).

Three meta-analyses (Zuckerman and Driver, 1985; DePaulo et al., 2003; Sporer and Schwandt, 2006) included preparation as a moderator and predicted that planning and rehearsal should facilitate deceptive performance by reducing cognitive/memory load. Although the meta-analyses yielded mixed results and weak effect sizes, planned messages were found to have shorter responses latencies and fewer silent pauses than unplanned ones. More recent research examining higher stakes deception has shown that fraud-relevant utterances were longer and more laden with details than non-fraudulent ones (Burgoon et al., 2015), a pattern duplicated by Braun et al. (2015) in their analysis of deceptive politicians’ messages. To the extent that detection accuracy is lower with planned than unplanned deception (Bond and DePaulo, 2006), some of that inaccuracy may be attributable to planned messages being indistinguishable from truth-telling. With advance preparation, communicators are better able to approximate normal, credible communication patterns.

### Recency of Target Incident or Issue

Depending on how distant it is, the time frame for requested narratives and accounts will have expectations associated with it for what is a complete, accurate, and clear response. Whereas recent events should impose equal recall difficulty on truth-tellers and deceivers, long-ago ones should be harder to recall for conscientious truth-tellers trying to be thorough and accurate than for deceivers fabricating a story or borrowing details from similar events. Some interview protocols like the cognitive interview capitalize on this reversal of expectations in which longer and more effortful answers should be associated with truth. Comparison questions in polygraph testing which are intended to create more mental conflict for truth-tellers than deceivers can be made even more challenging when the time frame is open-ended. The question, “Have you ever lied to someone who trusted you?” may prompt truth-tellers to ponder and hesitate more than deceivers. Other aspects of cognitive work unique to deceivers are the activation of executive memory to make the decision to lie, the construction and selection among possible lies and the comparison to the truth, which may guide decisions about which form and content of the lie is likely to be the most efficacious.

### Cooperative-Adversarial Relationship

Intertwined with the genre of discourse is whether the relationship between communicators constitutes a cooperative or adversarial one. Grice (1989) proposed that communicators enter encounters with a presumption of cooperativeness. In practice, however, many communication contexts and relationships are recognized as adversarial–criminal interrogations, litigation, labor disputes, negotiations, dispute mediations, and divorce proceedings that place the parties at odds with one another, among others–during which the assumption of cooperativeness is suspended. In adversarial interactions, one cannot even assume that interlocutors are using language in the same way. For example, in organizational contexts, management may practice strategic ambiguity as a way to reduce rather than facilitate understanding.

In other cases, participants with hidden agendas may wish to give the appearance of cooperativeness while covertly violating the Gricean maxims (McCornack, 1997). Under these circumstances the success of the deception will depend on how clandestine the deceit is. Predictions about how much cognitive difficulty is associated with lying should take into account how much cognitive “work” is needed to keep nefarious motives hidden. Unwitting interlocutors, for example, may lessen the difficulty for deceivers by proposing plausible explanations for a sender’s otherwise implausible response, thereby helping deceivers construct a believable narrative as a dialog unfolds.

### Relational Familiarity

Buller and Burgoon (1996b) identified three types of familiarity, one of which is relational familiarity. People who are well acquainted with one another have prior knowledge and a history of behavior against which to judge anything that is said. For the deceiver, this can make devising a plausible lie that evades detection more challenging inasmuch as there are numerous touchpoints against which the deceiver must make mental comparisons before actually uttering the lie. At the same time, deceivers can capitalize on their familiarity with the receiver to adapt lies more specifically to the interlocutor’s knowledge bank and can watch the receiver for telltale signs of disbelief. Buller and Aune (1987) found deceivers interacting with familiar others successfully restored their original level of animation, while deceivers interacting with strangers became less immediate and animated over time. Thus, deceivers took advantage of their relationship to improve their performance over time. Burgoon et al. (2001) found similar results in that deceivers interacting with friends rather than strangers were better able over time to manage their informational content, speech fluency, non-verbal demeanor, and image. Presumably the improved performances were accompanied by a corresponding reduction in cognitive difficulty for deceivers relative to truth-tellers. Since receivers seldom expect to be lied to, relational familiarity probably confers more of an advantage on the sender than the receiver.

### Communication Unit of Analysis

The sampling unit for deception research and meta-analyses typically has been the single utterance, turn at talk, or answer to a single question. Such samples may be less than 30 s in length. Yet deception may be woven into a series of utterances (e.g., an interview), interpenetrate an entire conversational episode, or span multiple conversations (e.g., multiple interrogations). The span of time from beginning to end of a deception event should affect how difficult it is to produce and maintain. Speculatively, as the number and duration of utterances related to an issue increases, the more cognitively challenging it should be to lie, inasmuch as one must remember what has been said previously, create consistency among utterances, reconcile what is being said with a potentially growing population of known facts, make decisions about which truthful details to divulge, decide what kinds of deception to enact, whether to change strategies (e.g., from concealment to equivocation), and so forth. Lengthy criminal justice interviews and interrogations depend on extended questioning to create more emotional and mental hardship for interviewees. Comparatively, producing brief utterances not only minimizes the amount of decision making, memory searching and message production demands that communicators incur (regardless of their veracity) but can also buy deceivers more time to concoct a credible response and to intersperse truthful details within one’s discourse to bolster believability.

The time course of the communication event thus may dictate its demand on cognitive and emotional resources. As the number of utterances or interchanges increases, demands on cognitive and emotional resources should increase differentially—up to an as-yet undetermined point. Beyond that, cooperative interactions should reduce the burden on deceivers by virtue of availing themselves of receiver feedback, making conversational repairs and meshing the dyad’s interaction patterns. We have witnessed this in several of our interviewing experiments. In one case, interviewees who were blindsided by unexpected questions initially gave non-fluent and improbable responses but with the aid of unwitting interviewers managed to spin out explanations that the interviewers accepted. Conversely, adversarial interactions such as interrogations may intensify the burden on deceivers. In drawing any conclusions, then, about whether lying is more difficult than truth-telling, it is necessary to specify the sampling unit for the respective truths and lies—short utterances or lengthy ones and single episodes or a series of them. Longer can be more difficult but may also introduce opportunities for countervailing repairs by deceivers.

## Implications

What are the implications of this decomposition of moderators of cognitive effort? First, the relationship between deception and cognitive effort is complex and highly variable. In some respects, the issue is one of definition of terms: What constitutes effort? If activation of more brain regions and processes constitutes effort, then deceit can be construed as creating greater *actual* cognitive work than truth. However, if effort requires some level of awareness, then only under more serious circumstances involving complex lies with significant (favorable or unfavorable) consequences may lying be *experienced* as more cognitively effortful.

Moreover, a variety of moderators can alter the deception-cognition relationship, and sometimes in contradictory ways. These previously unidentified or untested moderators may account for the oft-times weak association between presumed cognitive effort and observable behavior. Only if the relevant influences can be parsed will it be possible to make sound and reliable cognition-based predictions and will cognition-based effects be replicable.

Also confounding the picture is that many factors like motivation and incentives exert similar influence on truth-tellers, thus making deceptive and truthful behavior patterns indistinguishable.

Too often, researchers have inferred backward from observable cues to likely cognitive causes, but such reasoning is fraught with indeterminacy due to the absence of single one-to-one correspondences between specific indicators and mental work. Even though more memory processes may be engaged, the observable indicators may not betray that work, they may arise from other causes, and they may be associated with both truth and deception.

Given these complicating factors, any cognitive load, cue-based approach may be difficult to utilize in practice. Only if the various moderators can be taken into account will such approaches be fully efficacious.

## Conclusion

This research topic on whether lying is more effortful cognitively than truth-telling is meant to challenge long-held assumptions. Challenging assumptions is clearly a worthwhile scientific endeavor, and this collection of essays will doubtless enlighten the issue while raising a number of salient considerations.

In the process of addressing this assumption, however, let us not erect false dichotomies, straw-man arguments, or extreme positions that produce more heat than light. For example, the assertions by McCornack et al. (2014) that the differences between truth and deception should all be attributed to memory and information processing is serious overstatement, just as their assertion that current models of deception impute too much cognitive work to deceptive message production is an overly broad gloss. As with so many issues surrounding human cognition and behavior, simple answers are facile but inaccurate and will set our science back. The typology of 13 moderators I have proposed derives from modeling deception as a communication phenomenon, the properties of which can exacerbate or alleviate cognitive demands. The non-exhaustive collection of moderators includes: (1) sender memory demands, (2) sender motivation, (3) incentives and consequences, (4) discourse genre, (5) form of prevarication, (6) expected response length, (7) sanctioning of the deceit, (8) communication medium, (9) advance preparation, (10) recency of the incident/issue, (11) relationship among interlocutors (e.g., cooperative or adversarial), (12) relational familiarity, and (13) size of unit of analysis. I invite further formalization and empirical testing by other deception scholars to disentangle the effects of these significant moderators.

## Conflict of Interest Statement

The author declares that the research was conducted in the absence of any commercial or financial relationships that could be construed as a potential conflict of interest.

## Footnotes

1 Space limitations do not permit developing the idea that deception may also instigate emotional work to regulate the kind of emotional flooding seen, for example, with escalating conflicts. But investigations of high levels of cognitive arousal may be well consider emotional correlates and regulatory overrides.

2 Although some meta-analyses have attempted to analyze the effects of communication context or genre on receiver detection accuracy (e.g., Bond and DePaulo, 2006; Hartwig and Bond, 2014), virtually no research has explicitly tested their effects on sender performance. Hartwig and Bond (2014), for example, had too few samples of different interview types to separate out different categories. Part of the challenge in deriving stable meta-analytic estimates is that only a small fraction of investigations have entailed interactions exceeding 1 min in length. Moreover, genre constructs such as interactivity are multidimensional. To test properly the effects of interaction on senders requires parsing the different attributes (e.g., participation, synchronicity, propinquity, multiplicity of modalities) and testing each independently to isolate the relevant features.

3 The least taxing form is concealment or omission in which deceivers simply leave out deceptive information. Although McCornack et al. (2014, p. 353) assert in IMT2 that “Zipf’s PLE [principle of least effort] compels speakers to minimize the total number of spoken words produced and shift instead toward objectively ambiguous language,” a claim consistent with the principle that humans are cognitively lazy, it fails to comport with the empirical evidence that people sometimes produce longer messages when deceiving than when telling the truth (e.g., Burgoon et al., 2014; Dunbar et al., 2014). Brevity, then, or effort is not the controlling factor.

4 It might be tempting to conclude that we can infer the degree of cognitive demands on senders by the accuracy with which their messages are detected by receivers. However, this would be a faulty inference inasmuch as detection accuracy is influenced by several factors other than sender performance (Burgoon et al., 2008; Burgoon, 2015). For example, deceivers may experience fewer cognitive demands under audio communication and yet inadvertently produce more telltale signs of deception due to lack of awareness or ability to manage the voice.

## References

[B1] Abe N. Greene J. D. (2014). Response to anticipated reward in the nucleus accumbens predicts behavior in an independent test of honesty. *J. Neurosci.* 34 10564–10572. 10.1523/JNEUROSCI.0217-14.2014 25100590 PMC6802594

[B2] Bereby-Meyer Y. Shalvi S. (2015). Deliberate honesty. *Curr. Opin. Psychol.* 6 195–198. 10.1016/j.copsyc.2015.09.004

[B3] Bond C. F. DePaulo B. M. (2006). Accuracy of deception judgments. *Pers. Soc. Psychol. Rev.* 10 214–234. 10.1207/s15327957pspr1003_2 16859438

[B4] Braun M. Van Swol L. M. Vang L. (2015). His lips are moving: pinocchio effect and other lexical indicators of political deceptions. *Discourse Process.* 52 1–20. 10.1080/0163853X.2014.942833

[B5] Buller D. B. Aune R. K. (1987). Nonverbal cues to deception among intimates, friends, and strangers. *J. Nonverb. Behav.* 11 269–290. 10.1007/BF00987257

[B6] Buller D. B. Burgoon J. K. (1996a). Another look at information management: a rejoinder to McCornack, Levine, Morrison, and Lapinski. *Commun. Monogr.* 63 92–98. 10.1080/03637759609376377

[B7] Buller D. B. Burgoon J. K. (1996b). Interpersonal deception theory. *Commun. Theory* 6 203–242. 10.1111/j.1468-2885.1996.tb00127.x

[B8] Buller D. B. Burgoon J. K. White C. Ebesu A. S. (1994). Interpersonal deception: VII. behavioral profiles of falsification, concealment, and equivocation. *J. Lang. Soc. Psychol.* 13 366–395. 10.1177/0261927X94134002

[B9] Burgoon J. K. (2015). Rejoinder to Levine, Clare et al.’s comparison of the Park–Levine probability model versus interpersonal deception theory: application to deception detection. *Hum. Commun. Res.* 41 327–349. 10.1111/hcre.12065

[B10] Burgoon J. K. Blair J. P. Strom R. (2008). Cognitive biases, modalities and deception detection. *Hum. Commun. Res.* 34 572–599.

[B11] Burgoon J. K. Buller D. B. (1996). Reflections on the nature of theory building and the theoretical status of interpersonal deception theory. *Commun. Theory* 6 311–328. 10.1111/j.1468-2885.1996.tb00132.x

[B12] Burgoon J. K. Buller D. B. (2015). “Interpersonal deception theory: Purposive and interdependent behavior during deceptive interpersonal interactions,” in *Engaging Theories in Interpersonal Communication, 2e*, eds Braithwaite D. O. Schrodt P. (Los Angeles, CA: Sage Publications), 349–362.

[B13] Burgoon J. K. Buller D. B. Floyd K. (2001). Does participation affect deception success? A test of the inter-activity effect. *Hum. Commun. Res.* 27 503–534.

[B14] Burgoon J. K. Buller D. B. Guerrero L. K. Afifi W. Feldman C. (1996). Interpersonal deception: XII. Information management dimensions underlying deceptive and truthful messages. *Commun. Monogr.* 63 50–69. 10.1080/03637759609376374

[B15] Burgoon J. K. Floyd K. (2000). Testing for the motivation impairment effect during deceptive and truthful interaction. *Western J. Commun.* 64 243–267. 10.1080/10570310009374675

[B16] Burgoon J. K. Langer E. (1995). “Language, fallacies, and mindlessness-mindfulness,” in *Communication Yearbook 18*, ed. Burleson B. (Newbury Park, CA: Sage Publication), 105–132.

[B17] Burgoon J. K. Marett K. Blair J. P. (2004). “Detecting deception in computer-mediated communication,” in *Computers in Society: Privacy, Ethics and the Internet*, ed. George J. F. (Upper Saddle River, NJ: Prentice-Hall), 154–166.

[B18] Burgoon J. K. Mayew W. J. Giboney J. S. Elkins A. C. Moffitt K. Dorn B. (2015). Which spoken language markers identify deception in high-stakes settings? Evidence from earnings conference calls. *J. Lang. Soc. Psychol.* 10.1177/0261927X15586792

[B19] Burgoon J. K. Nunamaker J. F. Jr. Metaxas D. (2010). *Noninvasive Measurement of Multimodal Indicators of Deception and Credibility*. Final Report to the Defense Academy for Credibility Assessment Tucson: University of Arizona.

[B20] Burgoon J. K. Proudfoot J. G. Wilson D. Schuetzler R. (2014). Patterns of nonverbal behavior associated with truth and deception: illustrations from three experiments. *J. Nonverb. Behav.* 38 325–354. 10.1007/s10919-014-0181-5

[B21] Burgoon J. K. Qin T. Hamel L. Proudfoot J. (2012). “Predicting veracity from linguistic indicators,” in *Paper Presented to the Workshop on Innovation in Border Control (WIBC) at the European Intelligence and Security Informatics Conference (EISIC)*, Odense.

[B22] Christ E. C. van Essen D. C. Watson J. M. Brubaker L. E. McDermott K. B. (2009). The contributions of prefrontal cortex and executive control to deception: Evidence from activation likelihood estimate meta-analyses. *Cereb. Cortex* 19 1557–1566. 10.1093/cercor/bhn189 18980948 PMC2693617

[B23] DePaulo B. M. Ansfield M. E. Bell K. L. (1996). lnterpersonal deception theory. *Commun. Theory* 6 297–310. 10.1111/j.1468-2885.1996.tb00131.x

[B24] DePaulo B. Kirkendol S. E. (1989). “The motivational impairment effect in the communication of deception,” in *Credibility Assessment*, ed. Yuille J. (Deurne: Kluwer), 51–70.

[B25] DePaulo B. M. Lindsay J. J. Malone B. E. Muhlenbruck L. Charlton K. Cooper H. (2003). Cues to deception. *Psychol. Bull.* 129 74–118. 10.1037/0033-2909.129.1.74 12555795

[B26] Dunbar N. E. Jensen M. L. Bessabarova E. Burgoon J. K. Bernard D. R. Robertson K. J. (2014). Empowered by persuasive deception: the effects of power and deception on interactional dominance, credibility, and decision-making. *Commun. Res.* 41 852–876. 10.1177/0093650212447099

[B27] Dunbar N. E. Jensen M. L. Burgoon J. K. Kelley K. M. Harrison K. J. Adame B. (2013). Effects of veracity, modality and sanctioning on credibility assessment during mediated and unmediated interviews. *Commun. Res.* 40 1–26.

[B28] Ennis E. Vrij A. Chance C. (2008). Individual differences and lying in everyday life. *J. Soc. Pers. Relat.* 25 105–118. 10.1177/0265407507086808

[B29] Fisher R. P. Geiselman R. E. (1992). *Memory-Enhancing Techniques for Investigative Interviewing: The Cognitive Interview.* Springfield, IL: Charles C Thomas.

[B30] Frank M. G. Feeley T. H. (2003). To catch a liar: Challenges for research in lie detection training. *J. Appl. Commun. Res.* 31 58–75. 10.1080/00909880305377

[B31] Ganis G. Kosslyn S. M. Stose S. Thompson W. L. Yurgelun-Todd D. A. (2003). Neural correlates of different types of deception: an fMRI investigation. *Cereb. Cortex* 13 830–836. 10.1093/cercor/13.8.830 12853369

[B32] Goldman-Eisler F. (1958). Speech analysis and mental processes. *Lang. Speech* 1 59–75.

[B33] Gougler M. Nelson R. Handler M. Krapohl D. Shaw P. Bierman L. (2011). Meta-analytic survey of criterion accuracy of validated polygraph techniques. *Polygraph* 40 194–305.

[B34] Grice H. P. (1989). *Studies in the Way of Words.* Cambridge, MA: Harvard University Press.

[B35] Hartwig M. Bond C. F. Jr. (2014). Lie detection from multiple cues: a meta-analysis. *Appl. Cogn. Psychol.* 28 661–676. 10.1002/acp.3052

[B36] Hopper R. Bell R. A. (1984). Broadening the deception construct. *Q. J. Speech* 70 288-302.

[B37] Horvath F. Blair J. P. Buckley J. P. (2008). The behavioural analysis interview: clarifying the practice, theory and understanding of its use and effectiveness. *Int. J. Police Sci. Manag.* 10 101–118. 10.1350/ijps.2008.10.1.101

[B38] Ito A. Abe N. Fujii T. Ueno A. Koseki Y. Hashimoto R. (2011). The role of the dorsolateral prefrontal cortex in deception when remembering neutral and emotional events. *Neurosci. Res.* 69 121–128. 10.1016/j.neures.2010.11.001 21074583

[B39] Kellermann K. (1992). Communication: inherently strategic and primarily automatic. *Commun. Monogr.* 59 288–300. 10.1080/03637759209376270

[B40] Knapp M. L. (2008). *Lying and Deception in Human Interaction.* Boston, MA: Allyn and Bacon.

[B41] Levine T. R. Shaw A. Shulman H. C. (2010). Increasing deception detection accuracy with strategic questioning. *Hum. Commun. Res.* 36 216–231. 10.1111/j.1468-2958.2010.01374.x

[B42] Mameli F. Mrakic-Sposta S. Vergari M. Fumagalli M. Macis M. Ferrucci R. (2010). Dorsolateral prefrontal cortex specifically processes general – but not personal –knowledge deception: multiple brain networks for lying. *Behav. Brain Res.* 211 164–168. 10.1016/j.bbr.2010.03.024 20307584

[B43] McCornack S. A. (1997). “The generation of deceptive messages: laying the groundwork for a viable theory of interpersonal deception,” in *Message Production: Advances in Communication Theory*, ed. Greene J. O. (Mahwah, NJ: LEA), 91–126.

[B44] McCornack S. A. Morrison K. Paik J. E. Wisner A. M. Zhu X. (2014). Information manipulation theroy 2 a propositional theroy of deceptive discourse production. J. Lang. and Soc. Psychol., 33 348–377. 10.1177/0261927x14534656

[B45] Miller G. R. Stiff J. B. (1993). *Deceptive Communication.* Thousand Oaks, CA: Sage Publcation.

[B46] Mullin D. S. King G. W. Saripalle S. K. Derakhshani R. R. Lovelace C. T. Burgoon J. K. (2014). Deception effects on standing center of pressure. *Hum. Mov. Sci.* 38 106–115. 10.1016/j.humov.2014.08.009 25278098

[B47] O’Hair H. D. Cody M. J. McLaughlin M. L. (1981). Prepared lies, spontaneous lies, Machiavellianism and nonverbal communication. *Hum. Commun. Res.* 7 325–339. 10.1111/j.1468-2958.1981.tb00579.x

[B48] Porter S. ten Brinke L. (2010). The truth about lies: what works in detecting high-stakes deception? *Legal Criminol. Psychol.* 15 57–75. 10.1348/135532509X433151

[B49] Rockwell P. Buller D. B. Burgoon J. K. (1997). The voice of deceit: refining and expanding vocal cues to deception. *Commun. Res. Rep.* 14 451–459. 10.1080/08824099709388688

[B50] Searcy W. A. Nowicki S. (2005). *The Evolution of Animal Communication: Reliability and Deception in Signaling Systems.* Princeton, NJ: Princeton University Press.

[B51] Shalvi S. De Dreu C. K. W. (2014). Oxytocin promotes group serving dishonesty. *Proc. Natl. Acad. Sci. U.S.A.* 111 5503–5507. 10.1073/pnas.1400724111 24706799 PMC3992689

[B52] Shalvi S. Eldar O. Bereby-Meyer Y. (2012). Honesty requires time (and lack of justifications). *Psychol. Sci.* 23 1264–1270. 10.1177/0956797612443835 22972904

[B53] Spence S. A. Farrow T. F. D. Herford A. E. Wilkinson I. D. Zheng Y. Woodruff P. W. R. (2001). Behavioural and functional anatomical correlates of deception in humans. *Neuroreport* 12 2849–2853. 10.1097/00001756-200109170-00019 11588589

[B54] Spence S. A. Kaylor-Hughes C. Farrow T. F. D. Wilkinson I. D. (2008). Speaking of secrets and lies: the contribution of ventrolateral prefrontal cortex to vocal deception. *Neuroimage* 40 1411–1418. 10.1016/j.neuroimage.2008.01.035 18308586

[B55] Sporer S. L. Schwandt B. (2006). Paraverbal indicators of deception: a meta-analytic synthesis. *Appl. Cogn. Psychol.* 20 421–446. 10.1002/acp.1190

[B56] Sporer S. L. Schwandt B. (2007). Moderators of nonverbal indicators of deception: a meta-analytic synthesis. *Psychol. Public Policy Law* 13 1–34. 10.1037/1076-8971.13.1.1

[B57] Tabatabaeian M. Dale R. Duran N. (2015). Self-serving dishonest decisions can show facilitated cognitive dynamics. *Cogn. Process.* 16 291–300. 10.1007/s10339-015-0660-6 26082072

[B58] ten Brinke L. Porter S. (2012). Cry me a river: Identifying the behavioral consequences of extremely high-stakes interpersonal deception. *Law Hum. Behav.* 36 469–477. 10.1037/h0093929 23205594

[B59] Turner R. E. Edgley C. Olmstead G. (1975). Information control in conversations: honesty is not always the best policy. *Kansas J. Speech* 11 69–89.

[B60] Vrij A. (2000). *Detecting Lies and Deceit: The Psychology of Lying and the Implications for Professional Practices.* West Sussex: John Wiley and Sons.

[B61] Vrij A. Fisher R. Mann S. Leal S. (2006). Detecting deception by manipulating cognitive load. *Trends Cogn. Sci. (Regul. Ed.)* 10 141–142. 10.1016/j.tics.2006.02.003 16516533

[B62] Vrij A. Granhag P. A. (2012). Eliciting cues to deception and truth: what matters are the questions asked. *J. Appl. Res. Mem. Cogn.* 1 110–117. 10.1016/j.jarmac.2012.02.004

[B63] Vrij A. Semin G. R. Bull R. (1996). Insight into behavior displayed during deception. *Hum. Commun. Res.* 22 544–562. 10.1111/j.1468-2958.1996.tb00378.x

[B64] Zuckerman M. DePaulo B. M. Rosenthal R. (1981). Verbal and nonverbal communication of deception. *Adv. Exp. Soc. Psychol.* 14 1–59. 10.1016/S0065-2601(08)60369-X

[B65] Zuckerman M. Driver R. (1985). “Telling lies: verbal and nonverbal correlates of deception,” in *Nonverbal Communication: An Integrated Perspective*, eds Siegman A. W. Feldstein S. (Hillsdale, NJ: Erlbaum), 129–147.

